# Artificial test-takers as transformed controls: measuring SAT difficulty drift and student performance

**DOI:** 10.3389/frai.2026.1692465

**Published:** 2026-03-16

**Authors:** Vikram K. Suresh, Saannidhya Rawat

**Affiliations:** University of Cincinnati, Cincinnati, OH, United States

**Keywords:** artificial intelligence, artificial test-takers, large language models, standardized testing, transformed control

## Abstract

**Introduction:**

Standardized test score trends are widely used to track student performance and inform policy, but they are difficult to interpret when exam content changes over time. We introduce an artificial test-taker framework that uses a fixed large language model as a stable benchmark to measure SAT Math difficulty drift and construct difficulty-adjusted measures of student performance.

**Methods:**

We built a longitudinal SAT Math item bank from SATs spanning 2007–2023. For each year, we generated 50 bootstrapped SAT forms that match the year-specific section blueprint and administered all items to GPT-4 under a fixed set of parameters and training to develop counterfactuals. We combine our difficulty benchmarks with national SAT Math scores released by the College Board to assess robustness to compositional changes.

**Results:**

The artificial test-taker framework indicates a statistically significant decline in SAT Math difficulty of 0.21σ relative to 2012. After adjusting for test difficulty using the transformed-control benchmark, student performance declines by 34 points in Average Difference in Scores (ADS) from 2012 to 2023. Heterogeneity analyses show that these declines are not uniform across racial groups.

**Discussion:**

Artificial test-takers provide a scalable, protocol-invariant audit of longitudinal comparability when traditional equating is infeasible, opaque, or incomplete. Our findings imply that evolving SAT Math content can mask substantial underlying performance decline and can differentially obscure trends across student subgroups. More broadly, transformed-control designs using AI offer a tool for benchmarking educational outcomes and for separating changes in measured performance from changes in the measurement instrument itself.

## 1 Introduction

The Scholastic Aptitude Test (SAT) has long been central in U.S. college admissions, and researchers often use SAT scores to study educational outcomes, inequality and labor market outcomes (Card and Rothstein, 2007; Hoekstra, 2009). Despite the wide usage of standardized tests—such as the SAT—as a key metric for tracking student preparedness over time, interpreting trends naively can be misleading if changes in exam difficulty are not accounted for as SAT itself is not a static instrument—its content and difficulty can evolve over the years. When such comparability fails, raw score trends can systematically overstate or understate changes in underlying performance. Large-scale testing programs attempt to preserve comparability through linking and equating: statistical procedures that place scores from different forms onto a common scale so that they can be interpreted interchangeably (Kolen and Brennan, 2014; Holland and Dorans, 2006). Since forms differ in difficulty, the relationship between raw and scaled scores must be adjusted so that a given scaled score reflects the same performance level regardless of test date. This is the principle behind the College Board's public description of equating for the SAT, which explicitly frames equating as ensuring that “a score …means the same regardless of when the student took the test” (College Board, 2019). In practice, however, equating and linking rest on strong requirements—most notably that forms measure the same construct and that the linking relationship is sufficiently invariant across populations and contexts—and these requirements can be stressed when tests and testing populations evolve (Michaelides, 2010; Guo et al., 2017).

A central threat to longitudinal comparability is *drift*: changes in items and construct representation over time. Even in well-run programs, items can become easier with exposure, change in ways that interact with curriculum and preparation, or shift in difficulty when the cognitive demands of the test evolve. Modern psychometric work therefore treats drift as an empirical object that must be monitored, using statistical detection approaches designed for repeated or continuous testing environments (Guo et al., 2017; Lee and Lewis, 2021; Kang, 2023). Importantly, drift can matter even when operational equating is performed, because equating can only correct difficulty differences under the assumptions that justify the linking relationship and under the information available such as anchor items, recycled questions, and the representativeness of the linking design.

These issues are especially salient for the SAT over the past decade. The test has experienced consequential design changes, including the 2016 redesign^1^ (College Board, 2016), while the surrounding admissions and participation environment has also shifted, particularly during the rapid expansion of test-optional policies in the COVID-19 era (Belasco et al., 2015; Bennett, 2021). At the same time, broader national indicators suggest that U.S. mathematics performance has declined in the pandemic period, including declines documented in NAEP long-term trend reporting (National Center for Education Statistics, 2023, 2024a,b). In this setting, interpreting SAT score trends requires separating at least two moving parts: (i) changes in the difficulty and construct representation of the SAT Math instrument, and (ii) changes in the performance and composition of the student population taking the test.

A practical obstacle is that researchers outside the testing agencies typically do not observe the operational equating and item-monitoring processes. Traditional equating designs rely on common anchor items or common examinees; these designs are difficult to implement for long-run audits when anchor items are proprietary and when it is challenging to administer historical forms to a stable sample of human examinees (Holland and Dorans, 2006). The common test-taker design is conceptually attractive because it reuses examinees rather than items, but it is rarely feasible at scale in high-stakes settings (Liu et al., 2025). As a result, applied research often treats SAT scores as directly comparable across years by assumption—an assumption that is convenient, but not always theoretically or empirically warranted when the instrument and participation context are changing.

In this paper, we introduce an artificial test-taker framework that uses a fixed large language model (LLM) as a stable benchmark to quantify SAT Math difficulty drift and to construct difficulty-adjusted measures of student performance. Conceptually, our design can be understood as a *fixed artificial test-taker equating audit*: instead of holding items constant via anchors, we hold the examinee constant by repeatedly administering year-specific SAT Math content to the same artificial participant under invariant testing conditions. This produces a benchmark series capturing how difficult each year's form would be for a constant “test-taker,” making it possible to distinguish changes in the instrument from changes in observed student scores. Methodologically, we then treat this benchmark as a transformed control, analogous to synthetic control approaches (Abadie and Gardeazabal, 2003), so that the difficulty-adjusted difference between student outcomes and the benchmark provides an interpretable measure of performance net of estimated difficulty drift (Callaway and Sant'Anna, 2021).

Our use of an LLM as the benchmark participant is motivated by two developments. The first is the rapid adoption of generative AI in assessment and educational measurement: recent literature argues that generative AI can serve as a diagnostic tool for item quality, validity evidence, and comparability, while also raising fairness and transparency concerns (Swiecki et al., 2022; Hao et al., 2024; Kaldaras et al., 2024; Bulut et al., 2024). The second is the emergence of methodological guidance for using LLMs as research objects or research participants, emphasizing the need for protocol specification, reproducibility, prompt transparency and careful reporting of model versioning and parameters (Chang et al., 2024; Abdelkarim et al., 2025; Zhao et al., 2025; Abdurahman et al., 2025). In line with these principles, we hold model version, prompting, and decoding fixed and treat each test-taker response as an independent interaction. We also conduct alignment and robustness analyses to assess whether model-perceived difficulty behaves in ways consistent with human difficulty signals and to evaluate concerns such as memorization.

Empirically, the artificial test-taker indicates a statistically significant decline in SAT Math difficulty of approximately 0.21 standard deviations relative to 2012. Accounting for this difficulty drift materially changes the interpretation of score trends. After difficulty adjustment, we estimate a larger decline in student performance: about 34 SAT Math points from 2012 to 2023. We interpret these as difficulty-adjusted observational differences, as our design controls for estimated test difficulty drift but cannot fully eliminate confounding from shifts in participation or student composition, which are particularly plausible in the test-optional era (Belasco et al., 2015; Bennett, 2021). Our heterogeneity analysis reveals that these difficulty-adjusted declines in student performance are not uniform across racial groups.

By making the comparability problem explicit and by utilizing an artificial test-taker when traditional equating is infeasible or incomplete, we contribute to literature in both educational measurement and applied AI that uses standardized scores for inference. More broadly, transformed-control designs using AI offer a tool for benchmarking educational outcomes and for separating changes in measured performance from changes in the measurement instrument itself.

The remainder of the paper is structured as follows. Section 2 describes the SAT data used in our study and the GPT-4 test-taking process. Section 3 outlines our empirical strategy, including the transformed control method. Section 4 presents our main findings on SAT difficulty and student performance trends along with heterogeneity analysis. Finally, Section 5 concludes with a discussion of the implications of our results for educational policy and future research.

## 2 Background and data

### 2.1 SAT questions

We constructed a question bank of SAT questions curated from a variety of online sources specializing in SAT preparation. These sources gave access to an extensive collection of past SATs. We gathered these tests in PDF format and stored them in a secure digital repository, encompassing a chronological collection from 2012 to 2023.

We transcribed each SAT exam PDF into a dataframe containing the question text, answer options for MCQs, source document, section and question numbers, question type, and calculator policy. MCQs present a set of possible answers, requiring the test-taker to select the most appropriate option. Answer type allowed a one-line input from the test-taker.^2^

Our final item bank contains 1,204 text-based questions from test forms spanning 2012–2023, representing 67% of all expected items. The remaining 33% of items contained visual elements incompatible with text-only models. Coverage is stable across years (range: 60%–73%), suggesting no systematic temporal bias in which items could be transcribed. This question bank was then used to administer multiple SATs to the LLM by bootstrapping the examinations for each year. In order to stabilize annual estimates, we ran 50 independent bootstraps per year under an identical prompt and decoding protocol; all API calls were stateless and seed-controlled to ensure independence across trials.

In 2016, the SAT underwent significant changes. The format shifted from a total score of 2,400 to 1,600, aligning with the pre-2005 SAT format. The Math section retained its value of 800 points. Importantly, the revision eliminated the penalty for incorrect answers, encouraging students to attempt all questions without fear of point deduction for incorrect guesses. Additionally, the Math section saw a reduction in the number of sections from three to two and an increase in questions from 54 to 58. Before 2016, calculators were allowed for all math sections. Starting 2016, the Math section included both a calculator-permitted and a *no calculator* section. It is important to acknowledge these format changes and how they might impact our study. For further details, refer to the Supplementary materials.

### 2.2 Student SAT score data

1. *National-level SAT scores data*. The College Board publishes yearly U.S. level reports summarizing SAT performance of the test-takers. The data from these reports include average scores, total test-takers and standard deviation in the scores for the mathematics test and the language and writing test. These reports are readily available year 2016 onwards, referred to as *post* period in our study, through the College Board website. For the years prior to 2016, which we refer to as the *pre* period, we collected the reports from the Internet Archive.

**Figure 1:**
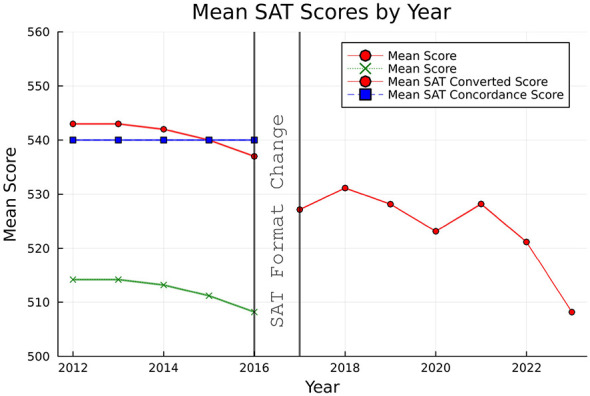
Average SAT scores of test-takers.

To account for the format change in the SAT exam in 2016, we used the concordance table provided by the College Board to convert the average SAT scores for the *pre* period to the *post* period. The concordance table provided by the College board is in multiples of 10. This requires rounding the average SAT score for exams in the *pre* period to the nearest multiple of 10 before it can be mapped to the average SAT score based on the concordance table. We used a linear model to interpolate the average SAT scores, which we call the SAT Converted scores. Figure 1 shows the average SAT score of students by year, before and after the conversion, as well as after using the linear interpolation. The green line represents the original average SAT scores for each year from 2012 to 2016. The blue line represents the converted score obtained after rounding the scores in the green line to the nearest multiple of 10, and then applying the conversion in the concordance table. The red line before the SAT format change represents the converted score using the linear interpolation to match the scores after the format change. The average SAT scores in red for each year from 2017 to 2023 remain unaltered and are directly available through the College Board reports. After the conversion, we can see that the mean SAT score decline in the *post* period compared to the *pre* period. Figure 1 suggests that students are doing worse in the *post* period exams than the *pre* period exams, which also includes a significant COVID-19 effect. However, this is under the assumption that the underlying exam did not change in difficulty.

2. *State-level SAT scores data*. For state-level SAT reports from 2016 onwards, we directly accessed the state-level reports available on the College Board's website. These reports provide comprehensive insights into the SAT performance of students on a state-by-state basis, enabling a detailed examination of trends and patterns in SAT scores across the United States. However, the availability of state-level SAT data prior to 2016 posed a unique challenge. To procure this historical data for the *pre* period (2012–2016), we used the Internet Archive. All human data are aggregate; no identifiable records were accessed; generative AI was used as an experimental agent, not as an author (separate disclosure provided).

3. *District-level SAT scores data*. We used publicly available school district level SAT data for the state of Massachusetts from the Massachusetts Department of Education. The data are available for all the school districts in the state for each year from 2004. The number of test-takers, the average reading, writing and mathematics scores are provided by school district for a given academic year. We identified 228 unique school districts commonly present over all the years considered in the study. For further details, refer to the Supplementary materials. We present two national views—(i) population-weighted national reports and (ii) a state unweighted average—and additionally a district-level panel (Massachusetts) to check robustness to composition.

### 2.3 Artificial participant

We used GPT-4-0125-preview as the artificial participant with fixed decoding (temperature = 0, max_tokens = 100); calls were executed in January–February 2024. Each year's evaluation comprised 50 bootstraps, each sampling a balanced SAT exam from our question bank from each year; outputs captured include final answer, correctness. Chain-of-thought was disabled, and answers were formatted deterministically for machine scoring. No further use of AI was made beyond this evaluation in the analysis.

## 3 Empirical strategy

In order to estimate the change in difficulty of the SAT math section relative to the baseline period, we extend the principles of Synthetic Control by Abadie and Gardeazabal (2003). LLMs like OpenAI's ChatGPT have revolutionized Natural Language Processing (NLP) tasks. Here, the transformed control is built by holding the test-taker fixed (GPT-4 under an identical protocol) and comparing year-to-year performance on contemporaneous items, so the counterfactual uses each year's own SAT content and precludes extrapolation.

We do not explicate the LLMs further, (Korinek 2023) and (Dell 2024) provide a comprehensive guide on their day-to-day use case. As these models are trained on increasing amounts of human generated text data and are continually improved, they become more adept at understanding and responding to queries. This progress opens new and sophisticated applications for these models. We highlight one such application within social sciences: what we refer to as transformed control in our research design. The LLMs have been used for direct causal reasoning by Kiciman et al. (2023), given a causal question to understand the models' capacity to accurately identify causal factors. Our approach is distinct from this procedure as we are using the LLM to generate control group data to enrich the inference about the student test-takers' performance. Although the control in this design does not serve for causal inference yet, it paves the way for incorporating modern AI tools into social science research.

The transformed control has similar advantages that are observed with synthetic control methods highlighted by (Cunningham 2021). Our method precludes extrapolation, the comparison with student test-takers' SAT outcomes is based on the LLM's performance in SAT questions from the same year. The counterfactual constructed relies on questions students themselves faced in that year. The LLM does not learn from previous attempts as each API call to the model is independent. This ensures the LLM's responses are not influenced by peeking at the student outcomes as each evaluation of the SAT is independent. Instead, it uses the knowledge in its training data to output an appropriate answer to the question provided. While standardized tests have been used to identify trends in student performance over time, it is assumed the underlying exam is uniformly challenging. Independently evaluating the standardized tests would require significant logistical undertaking. The LLM can instead act as the control and thus bridge the gap between qualitative and quantitative research. In our experiment the LLM by OpenAI, GPT-4 is the control unit.

### 3.1 Scoring methodology

**Figure 2:**
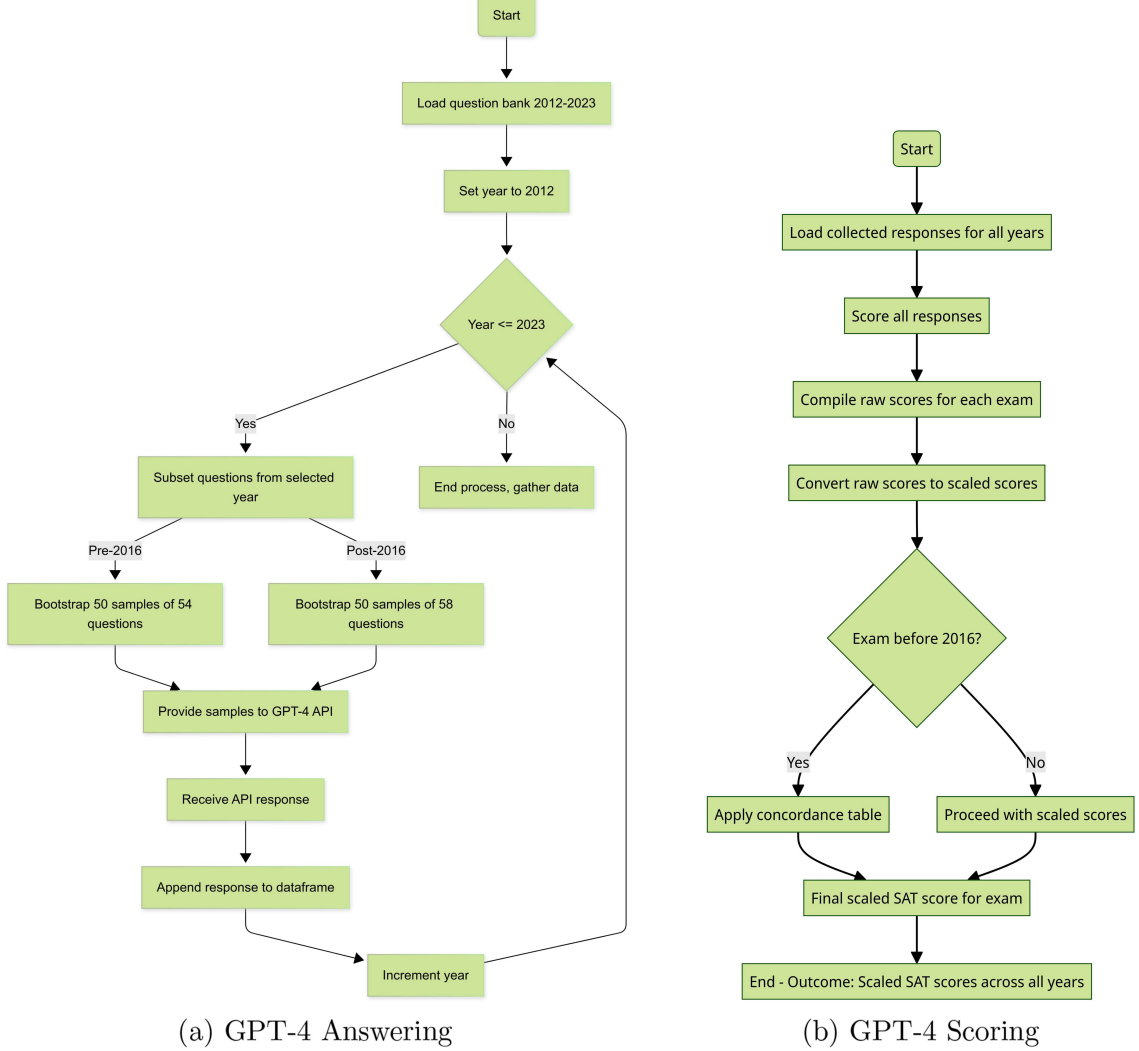
GPT-4 data processing flowcharts. **(a)** GPT-4 answering. **(b)** GPT-4 scoring.

The section outlines the methodology employed to enable OpenAI's models to answer SAT questions. As illustrated in Figure 2a, we randomly sampled 50 SATs for each year from the question bank using bootstrapping while maintaining identical number of question types as per the SAT format of the given year. We then prompted GPT-4 by OpenAI to take each of the 50 sampled exams for each year. Then, we calculated the number of correct answers and their proportion (number of correct answers divided by total questions) to assess the model's performance and compared the distributions obtained for different periods. We keep model version and decoding fixed across all calls and treat each API call as stateless and independent, ensuring protocol invariance across years.

First, we sample an SAT without replacement from our SAT Math Question bank as in Section 2. Next, we proceed by selecting individual questions from the sampled SAT exams, each of which is then presented to the GPT-4 model using a carefully pre-formulated prompt. The structure of our prompt has been provided in the Supplementary materials. Once GPT-4 receives this API call, it provides a response in a format specified by our prompt. The answer is then saved to a dataset that keeps a record of all the questions that have been answered and their corresponding answers. This process continues until all the questions in the sampled exam have been exhausted. All the questions and answers associated with each exam are compiled into a dataset. Once all the generated answers are collected, we convert raw score to scaled SAT score and recenter for accurate comparison over time, as described in Figure 2b and explained in Section 2.2.

The College Board accounts for variation in exam difficulty before scoring the students' performance within that year. As some students are likely to receive relatively more difficult exams than their peers taking the exam in the same year, the final score factors in the difficulty level to allow the accurate assessment of students relative to students in the same peer group. The process of bootstrapping as described generates exams within the year that include questions of varying difficulty, mimicking the exams received by student test-takers. Thus, some sampled exams are more difficult than others. Thereby, ensuring we evaluate the average SAT for a given period.

#### 3.1.1 Prompting

We employed a zero-shot prompt, meaning no example solutions were provided to the LLM to facilitate answering the question. Further, we did not allow for chain-of-thought. The LLM was proscribed from sequential reasoning. We use the same model build [gpt-4-0125-preview] and unchanged prompting/decoding parameters for all years evaluated. The question was provided through the prompt and the LLM was asked to provide one character letter output for multiple choice questions and the appropriate numerical or equation output for the answer type questions. The prompting strategy remained identical over all periods, ensuring no bias through the prompt. Since all prompts are independent API calls to the model, the LLM had no memory of previous questions. The LLM therefore is tasked to answer each question independent of any other question or reference to the period from which the question is gathered. This ensured the comprehensive evaluation of the difficulty of the questions from appropriate periods. Structured outputs to refine the LLM response were not available at the time of the study. Further prompting details and GPT-4 model parameters are provided in the Supplementary materials.

#### 3.1.2 Question embeddings and difficulty alignment

To ensure that the composition of SAT questions in our study accurately reflects the intended distribution of difficulty levels, we perform a difficulty alignment analysis to ensure no over- or under-representation of any particular difficulty type. Further, having identified the difficulty of the questions across different years, we can assess the performance of the LLM on questions of varying difficulty. Below, we describe the methodology used and the results of this analysis.^3^

For the exam years before 2014, the College Board provided difficulty ratings for each question. These difficulty ratings were not provided by the College Board for exams starting 2014, hence we predicted the difficulty rating using a machine learning model. To avoid temporal leakage, training uses only preserved 2008–2011 items and pre-2014 labels; 2015+ items are prediction-only and never used for training. The difficulty rating of the question for the students was between 1 and 5, with 1 being the easiest and 5 being the hardest. We used these ratings to study whether LLM's concept of difficulty aligns with the student test-takers. For this task, we used the text-embedding-3-large model from OpenAI (Neelakantan et al., 2022). Embeddings are a way to represent text as a vector in high dimension, which contains the semantic meaning of the text. These embeddings show how the LLM identifies the text in its internal representation and has been used for various language processing tasks (Xue et al., 2018; Gao et al., 2019).

The embeddings created by the model are used as features to predict the difficulty of the questions. In addition, the SAT was a pencil and paper test for the years considered in this study, and the difficult questions appear at the end of each section. We use this information as an additional feature in the classification model by including a progress bar as a numerical representation of the progress through the section. To simplify the classification task, we group difficulty ratings 1 and 2 as easy (310 questions), 3 as medium (265 questions), and 4 and 5 as hard (205 questions). The model is trained on 80% of the data having balanced the classes and tested on the remaining 20% of data. We also balance classes and report out-of-sample performance on the held-out 20% split to verify alignment. Using a Random Forest Classifier, the model achieves an accuracy of 0.789 on the testing data. Additional details regarding the confusion matrix for the classification are available in Supplementary materials. The model is then used to predict the difficulty of the questions in the SAT from 2015 to 2023.

**Figure 3:**
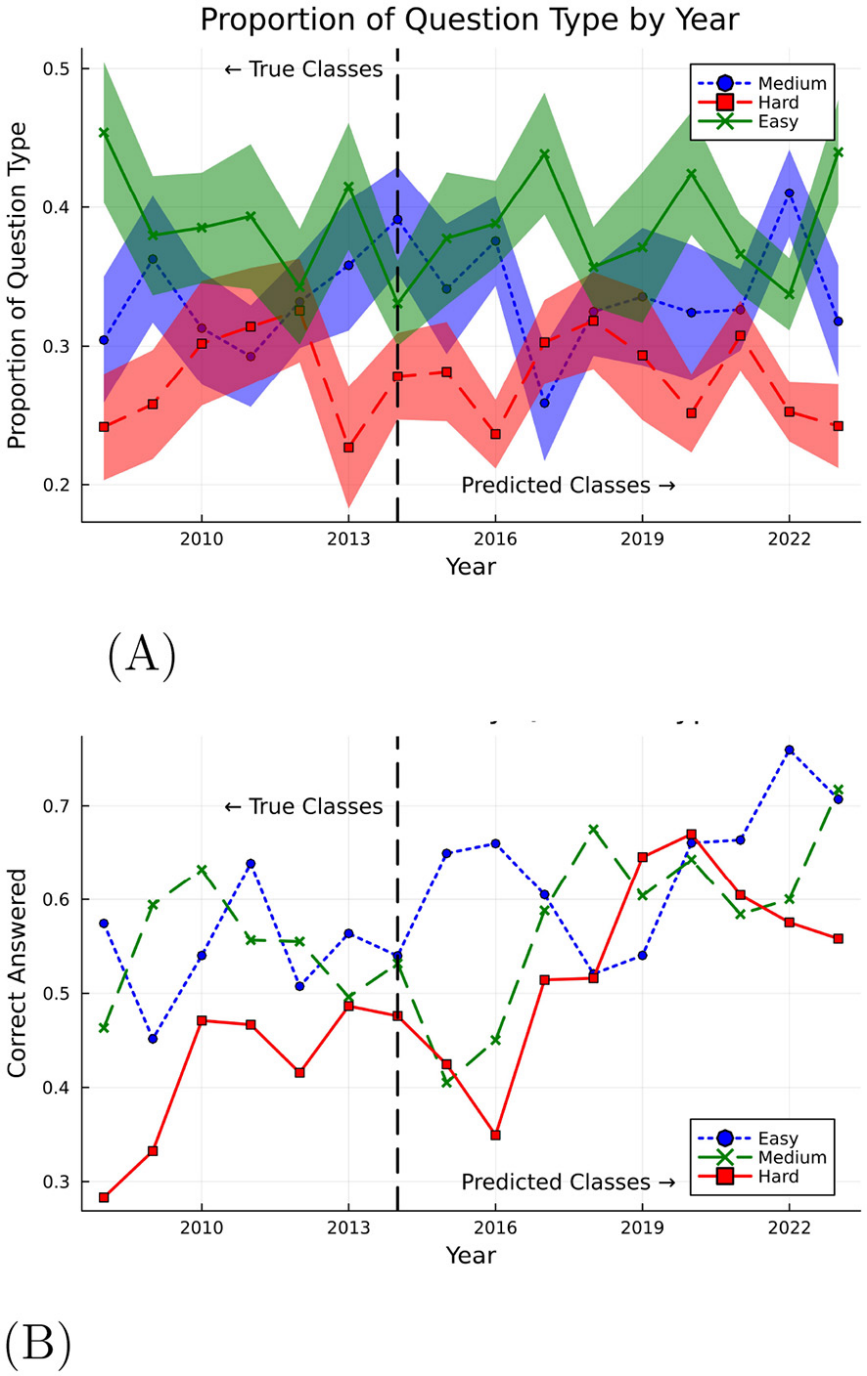
Question difficulty alignment. **(A)** SAT difficulty consistency. **(B)** Accuracy rate by question difficulty.

Figure 3A displays the results, and highlights the proportion of easy, medium, and hard questions in the bootstrapped SATs from each year. Further, the average proportion of easy, medium, and hard questions in the complete SATs gathered for the years 2008 to 2014 were 40%, 34% and 26% respectively. The proportion of easy, medium, and hard questions in the bootstrapped SAT approximates the average proportion in the complete SATs, suggesting that years 2015 onwards had similar proportion of easy, medium, and hard questions when juxtaposed against the preceding years. These results suggest that SATs from varying years had similar proportions of easy, medium, and hard questions. This is important as it allows us to compare the performance of the LLM across years without worrying about the composition of the SATs.

Having predicted the difficulty of the questions, we use the predicted classes to identify the change in performance of the LLM, i.e., the proportion of each question type LLM is correctly answering over time. In Figure 3B, we show the correct response rate of the LLM for easy, medium, and hard questions. First, we notice the LLM performs better on easy and medium difficulty questions compared to hard questions for the years 2008 to 2014. The LLM's lower accuracy rate on questions rated difficult for student test-takers suggests a potential alignment in perceived difficulty between students and the LLM. Additionally, while performance improves across all difficulty levels over time, the most pronounced gains are observed for hard questions.

### 3.2 Reduced form

For this study, we have two parameters of interest. First, we want to estimate the change in difficulty of SAT math section. Second, after controlling for exam difficulty, we want to estimate the change in performance of test-takers. We explain our strategy to estimate each of these parameters below. We fix 2012 as baseline and report changes relative to that year for both the model and students, so that the Average Difference in Scores (ADS) compares like-for-like movements from a common starting point.

1. *Transformed control*. To estimate the change in difficulty of the SAT, we formally consider the question bank *Q* of 1,204 SAT questions from the years 2012 to 2023. *S*_*t*_ is a collection of subsets of *Q* and each *S*_*t*_ contains randomly sampled exams from the question bank *Q* without replacement, ensuring unique questions populate each exam. Each exam *E*_*j, t*_ ∈ *S*_*t*_ and each *E*_*j, t*_ ⊂ *Q*, where *j* = 1, 2, ..., 50.

∀ *E*_*jt*_ ∈ *S*_*t*_, with *t* = {2012, 2013, ..., 2023} and where *q*_*ijt*_ is a question in exam *E*_*jt*_, we get


$$n_{mjt}=\overset{\left|E_{jt}\right|}{\underset{i=1}{\sum }}\chi_{A_{q_{ijt}}}\left(f_{m}\left(q_{ijt}\right)\right)\text{ and }p_{mtj}=\frac{n_{mjt}}{\left|E_{jt}\right|}\text{,}$$

*n*_*mjt*_ is the number of questions answered correctly by model *m*, for exam *j* in period *t*. *p*_*mjt*_ is the proportion of questions answered correctly by model *m* ∈ {GPT-4 Turbo, Claude 3.5}, for exam *j* in period *t*. While we restrict this study to GPT-4 model, we include results from other models in the Supplementary materials. |*E*_*jt*_| is the cardinality of *E*_*jt*_, which in our paper represents the number of questions in exam *E*_*jt*_. *f*_*m*_ is some LLM based function that takes in a question as input and provides an answer as output, depending on whether the answer is for MCQ or answer-type question. χ_*A*__*q*__*ijt*___ is a characteristic function that is 1 if output from *f*_*m*_ is correct and 0 otherwise, depending on a set of correct answers *A*_*q*_*ijt*__ for question *q*_*ijt*_.

From these performance evaluation measures *n*_*mjt*_ and *p*_*mjt*_, we obtain the statistics μ_*mt*_ and ν_*mt*_, along with their respective standard errors σ_μ*mt*_ and σ_ν*mt*_ -


$$\mu_{m,t}=\frac{1}{\left|S_{t}\right|}\overset{\left|S_{t}\right|}{\underset{j=1}{\sum }}n_{mjt}$$

$$\nu_{m,t}=\frac{1}{\left|S_{t}\right|}\overset{\left|S_{t}\right|}{\underset{j=1}{\sum }}p_{mjt}$$

μ_*mt*_ is the mean number of questions answered correctly by model *m* in period *t*. ν_*mt*_ is the mean proportion of questions answered correctly by model *m* in period *t*. |*S*_*t*_| is the cardinality of the set *S*_*t*_, which is 50 for all time periods.

2. *Performance comparison*. Our second parameter of interest is the change in mathematical performance of the test-takers. To estimate this parameter, we utilize the estimation procedure from multi-period difference-in-difference framework as expounded in (Callaway and Sant'Anna 2021). Next, we explain how we borrow ideas and notation from (Callaway and Sant'Anna 2021) to fit our empirical setting.

The LLM, GPT-4 is a fixed entity given the training and prompting technique are held constant. If the test-takers did not change in composition, the expected change in performance of the students in the SAT exam is the estimated change in the underlying exam difficulty. We use the potential outcome notation for the test-takers' SAT score measurements, *Y*_*i, t*_(0) is potential *i*^*th*^ measurement in period *t* for the unchanging test-takers. While *Y*_*i, t*_(*g*) is the actual outcome measured for students. We formally define the *j*^*th*^ exam score for the LLM taken in period *t* as *T*_*j, t*_. This allows us to construct a strong parallel trends assumption as the following,


$$\begin{array}{l} 𝔼\left[Y_{t}\left(0\right)-Y_{t-1}\left(0\right)\right]=𝔼\left[T_{t}-T_{t-1}\right],\text{where}2012<t\leq 2023. \end{array}$$

Since our transformed control is the unobserved parallel trend, the estimation mimics treatment effect estimation in a classical difference-in-difference approach without making causal claims. Additionally, under this parallel trends like assumption, the expected change in the LLM's score is the estimated change in difficulty of the SAT exam between the measurement periods. We borrow some notation from the estimation procedure described by (Callaway and Sant'Anna 2021) and adopt for our use case. Our control provides a richer perspective about the difference in student performance, we refer to this estimand as the Average Difference in Scores (ADS).


$$\begin{array}{l} ADS\left(\hat{t},t\right)=𝔼\left[Y_{t}\left(g\right)-Y_{t}\left(0\right)\;|\;\hat{t}\;\right] \end{array}$$

In the above equation, *Y*_*t*_(0) is the unobserved potential outcome. However, when we impose strong parallel trends, we can identify the ADS with reference to a baseline year,


$$\begin{array}{l} ADS\left(\hat{t},t\right)=𝔼\left[Y_{t}\left(g\right)-Y_{t}\left(0\right)\;|\;\hat{t}\;\right]+𝔼\left(Y_{\hat{t}}\left(0\right)|\hat{t}\right)-𝔼\left(Y_{\hat{t}}\left(0\right)|\hat{t}\right)\\ \;=𝔼\left[Y_{t}\left(g\right)-Y_{\hat{t}}\left(0\right)|\hat{t}\right]-\left(𝔼\left[Y_{t}\left(0\right)-Y_{\hat{t}}\left(0\right)|\hat{t}\right]\right) \end{array}$$

Now, notice that because we start at the same baseline, we have $Y_{\hat{t}}\left(0\right)=Y_{\hat{t}}\left(g\right)$. So,


$$\begin{array}{l} ADS\left(\hat{t},t\right)=𝔼\left[Y_{t}\left(g\right)-Y_{\hat{t}}\left(0\right)\;|\;\hat{t}\;\right]-𝔼\left[Y_{t}\left(0\right)-Y_{\hat{t}}\left(0\right)\;|\;\hat{t}\;\right]\\ \;=𝔼\left[Y_{t}\left(g\right)-Y_{\hat{t}}\left(g\right)\;|\;\hat{t}\;\right]-𝔼\left[Y_{t}\left(0\right)-Y_{\hat{t}}\left(0\right)\;|\;\hat{t}\;\right]\\ \;=𝔼\left[Y_{t}\left(g\right)-Y_{\hat{t}}\left(g\right)\;|\;\hat{t}\;\right]-𝔼\left[T_{t}-T_{\hat{t}}\;|\;\hat{t}\;\right] \end{array}$$

The above expression is identifiable as all the terms are observed. Hence, using strong parallel trends like assumption and fixing the baseline at some $\hat{t}$, we can estimate ADS.

Our analysis is operationalized through a standard parametric linear regression model that accommodates multi-valued discrete treatment variable, which can be represented by the following regression equation:


$$\begin{array}{l} \text{Δ}Z_{i,t,s}=\overset{2023}{\underset{t=2013}{\sum }}\text{1}\left\{\tau_{i}=t\right\}\gamma_{t}+\overset{2023}{\underset{t=2013}{\sum }}\text{1}\left\{\tau_{i}=t\right\}\times \text{1}\left\{s=student\right\}\beta_{t}+\epsilon_{i,t,s} \end{array}$$

In our study, Δ*Z*_*i, t, s*_ is the change in SAT score for unit i from the baseline year 2012, where *s* ∈ {*student, LLM*} and τ_*i*_ is the exam year of unit i. The parameter β_*t*_ is the $ADS\left(\hat{t},t\right)$ measuring expected change in test-takers' math performance relative to baseline 2012 controlling for the exam difficulty through the LLM.

## 4 Results

We first present the performance of GPT-4 in the SATs over the years. Next, we use GPT-4's performance as a control to estimate the changes in student performance.

### 4.1 Change in difficulty of SAT math section

In evaluating GPT-4's performance on SAT math sections, we accounted for the varying number of questions in the *pre* format change (54 questions) and *post* format change (58 questions) periods. To enable a fair comparison, we also analyzed the proportion of correctly answered questions for each year, i.e., the ratio of number of correct answers to total questions per exam over 50 bootstrap samples. This approach ensures a normalized comparison despite the different total question counts. This normalization lets us interpret year-to-year differences as changes in form difficulty rather than artifacts of question counts.

#### 4.1.1 Average performance of LLM on SATs

**Figure 4:**
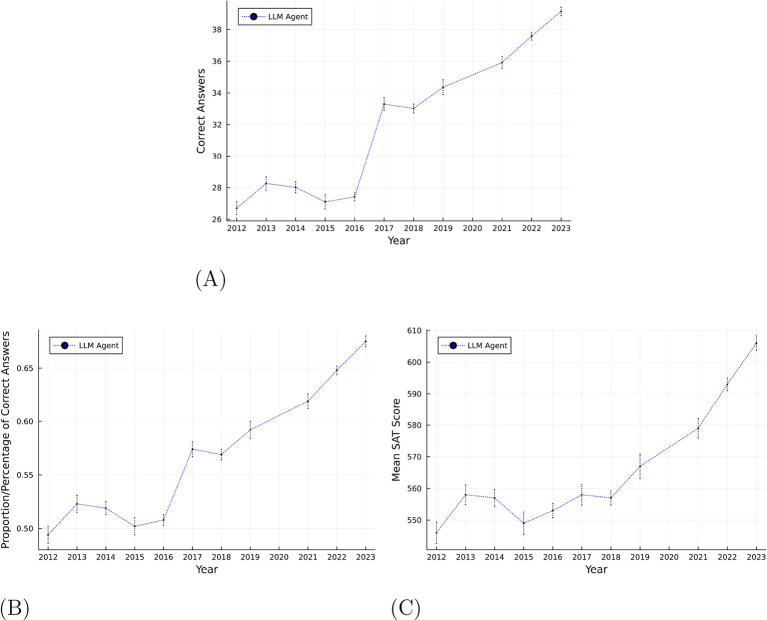
Performance of LLM on SAT exams over the years. Each point represents the mean across 50 bootstrap samples per year. Shaded regions show 95% bootstrap confidence intervals (2.5th–97.5th percentiles). **(A)** Shows raw correct answers, **(B)** shows proportion correct, and **(C)** shows scaled SAT scores (converted to post-2016 scale for years prior to 2017). **(A)** Number of questions correctly answered by LLM in SATs from each year. **(B)** % of questions correctly answered by LLM in SATs from each year. **(C)** Scaled SAT scores for LLM in SATs from each year.

Figure 4A shows the raw number of questions correctly answered by the LLM in the SATs from each year. The LLM's performance is found to be increasing over time. As there are 4 additional questions after the format change, it is challenging to interpret the raw number of questions correctly answered. Thus, Figure 4B provides the corresponding percentage of questions correctly answered by the LLM in the SATs from each year. Figure 4C provides the scaled SAT score which can be benchmarked against maximum SAT score of 800. We use the concordance tables provided by the College Board to recenter the scores before the format change as described in Section 2. As shown, GPT-4's performance is increasing over time. Since 2012, the SAT has been declining in difficulty by roughly 4 points per year.

#### 4.1.2 Dispersion over the years

We also analyze the dispersion of GPT-4's performance relative to the average student performance in 2012. This analysis provides insights into how the LLM's performance has evolved over time compared to the average student test-taker. Specifically, we compared the performance of the LLM on the SAT to the national student score distribution from 2012, using this fixed year as a baseline to assess the LLM's relative standing. Additionally, we performed a year-by-year comparison, evaluating the LLM's performance relative to the national student score distribution for each corresponding year. In both analyses, we quantified the gap between the LLM and the average student in terms of the standard deviation of student scores, providing a standardized measure of the difference in performance over time.

**Figure 5:**
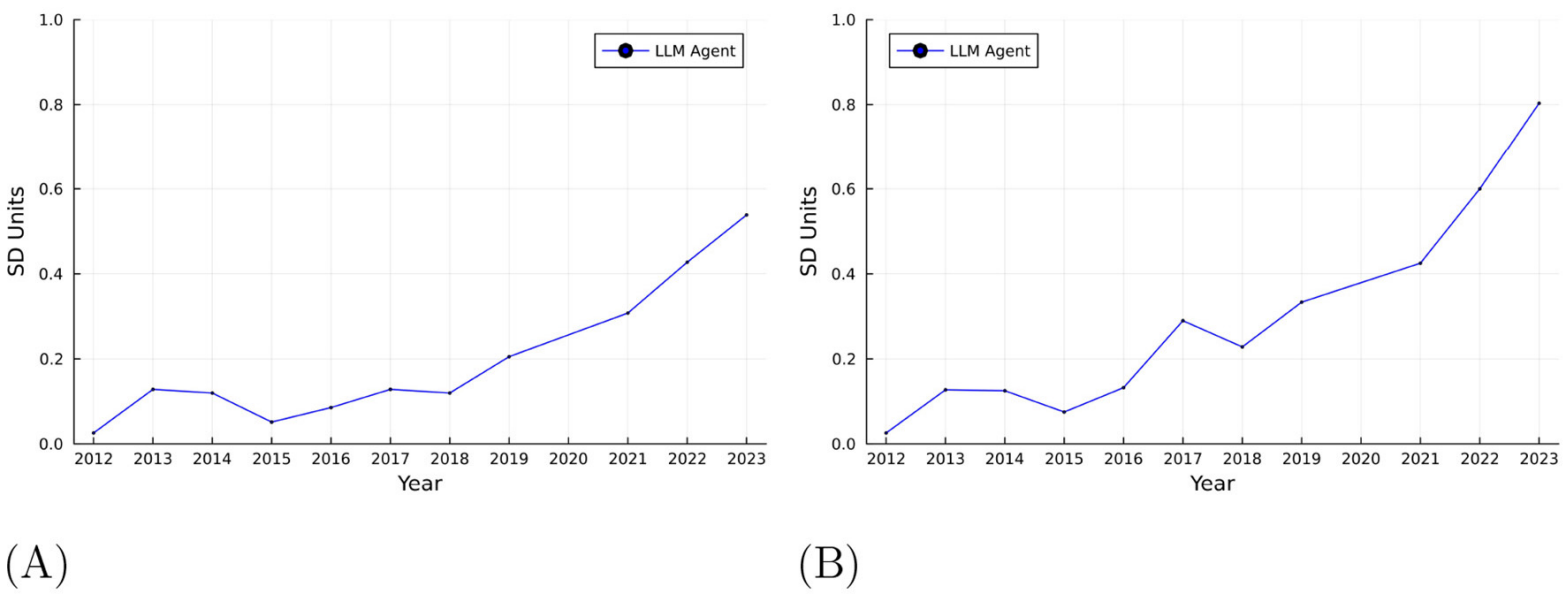
GPT performance relative to student scores. **(A)** GPT performance relative to student score in 2012. **(B)** GPT performance relative to Student Score for each year.

Figure 5A shows the performance of the LLM relative to the student score distribution in 2012. As shown by this figure, the LLM's average performance is deviating further away from the average student performance over the years, and moving closer to the right tail of the student score distribution.^4^ In 2012, the gap between the LLM and the average student was 0.02σ. However, by 2023, this gap increased to 0.6σ, and the average over 12 years is 0.21σ relative to the base year of 2012. These standardized gaps provide effect-size comparability and are computed using the 2012 student SD (117) as noted.

Figure 5B shows the performance of the LLM relative to the student score distribution for each year.^5^ The gap between the LLM and the average student is also increasing over time and even more pronounced, indicating that the LLM's performance is diverging from that of the average student. In 2012, the gap was 0.02σ, and by 2023, it had increased to 0.8σ, with an average of 0.32σ over the 12 years.

### 4.2 Change in student performance

We begin by providing results for estimated $ADS\left(\hat{t},t\right)$ for the baseline year 2012. This gives us the relative change in the performance of average student test-taker, having controlled for the SAT difficulty using transformed control. We perform this comparison at the national level using both the population performance data provided by the College Board and the state-by-state unweighted average SAT scores to replicate a representative national sample. For the state of Massachusetts, we use the school district-level SAT scores to estimate ADS.

**Figure 6:**
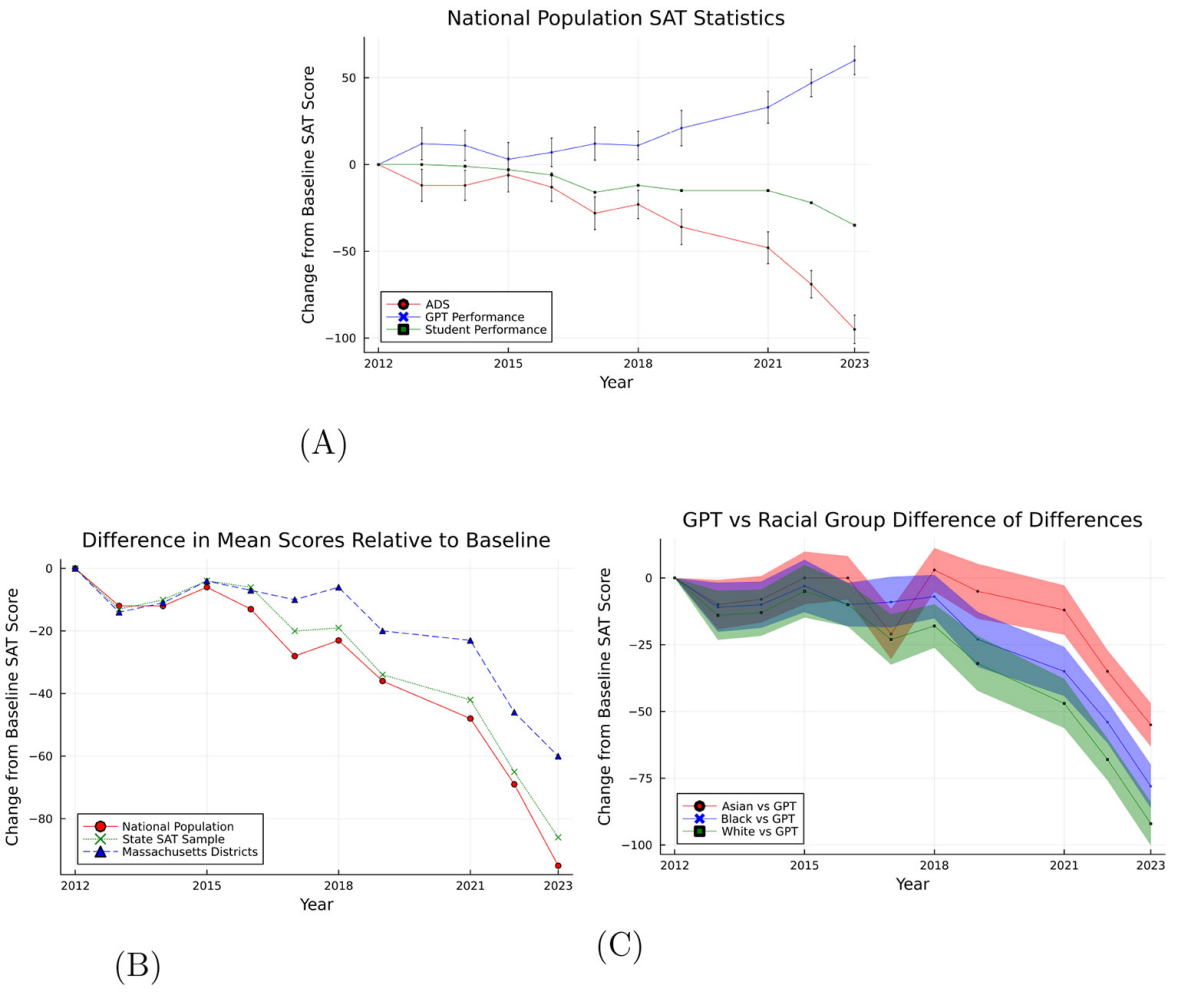
Estimated Average Difference Scores (ADS) relative to 2012 baseline. ADS = (GPT-4 change from baseline) − (Student change from baseline), measuring difficulty-adjusted student performance change. **(A)** Uses national population data (*n* > 1.5 million test-takers per year). **(B)** Compares national population, state unweighted average (*n* = 51 states), and Massachusetts district panel (*n* = 228 districts). **(C)** Shows heterogeneity by race. Error bars represent 95% confidence intervals from bootstrap simulation (100,000 draws from t-distributions). **(A)** Estimated $ADS\left(\hat{t},t\right)$, national population. **(B)** Estimated $ADS\left(\hat{t},t\right)$. **(C)** Estimated $ADS\left(\hat{t},t\right)$ for Asian, black and white students.

Figure 6A shows the estimated ADS, which gives us decline in SAT scores of student test-takers after controlling for exam difficulty. From this figure, we notice that the average performance of the test takers declined by 34 points from 2012 to 2023. Table 1 summarizes the main results: GPT-4's score increased by 59.8 points (SE = 4.68) from 2012 to 2023, while student scores declined by 35 points (SE = 0.13), yielding an ADS of 94.8 points (SE = 4.68). This indicates that the difficulty-adjusted decline in student performance is substantially larger than the raw score decline suggests.

**Table 1:** Main results: GPT-4 and student SAT math performance (2012–2023).

**Year**	**GPT-4 score**	**(SE)**	**Student score**	**(SE)**	**ADS**	**(SE)**
2012	546.2	(3.40)	543	(0.09)	0.0	—
2016	553.0	(2.27)	537	(0.10)	12.8	(4.09)
2020	597.2	(3.16)	523	(0.08)	71.0	(4.64)
2023	606.0	(3.21)	508	(0.09)	94.8	(4.68)
*Change (2012 → 2023)*						
	+59.8	(4.68)	−35	(0.13)	+94.8	(4.68)

GPT-4 scores from 50 bootstrap samples per year (SE = SD/$\sqrt{50}$). Student scores are national population means converted to post-2016 scale. ADS = (GPT-4 change) − (Student change), relative to 2012 baseline.

We used the state-by-state unweighted average SAT scores to construct a representative national sample along with the national population data provided by the College Board. The ADS using both samples are shown in Figure 6B along with the district-level scores for the state of Massachusetts. Figure 6C shows the estimated ADS for Asian, Black and White students. White students show the largest decline in average performance of 33 SAT points, while Black and Asian students declined by 25 and 15 points. The College Board reports do not have consistent demographic information over the period considered for Hispanic students, we therefore considered the most consistent demographic breakdown of the SAT scores present in the report.

These results highlight key trends; state-by-state unweighted SAT data generally mirrors national population trend. Massachusetts presents a slightly different narrative, with changes in SAT scores that often align with national trends but with some exceptions. For instance, the decline in Massachusetts is less pronounced in certain years, indicating possible state-specific factors that might have cushioned the impact. Overall, we find a notable difference in performance of the LLM and the performance of test takers, with LLM's performance improving and student performance declining over time. These estimates are qualitatively invariant between the national population, the state unweighted average, with Massachusetts serving as a district-panel check that largely follows the national trend. Taken together, a steady easing in form difficulty coupled with a difficulty-adjusted decline in student performance underscores the value of an artificial test-takers' benchmarking when comparing cohorts over time.

### 4.3 Robustness checks

We conduct several robustness checks to address potential concerns about our methodology.

#### 4.3.1 Memorization test

**Figure 7:**
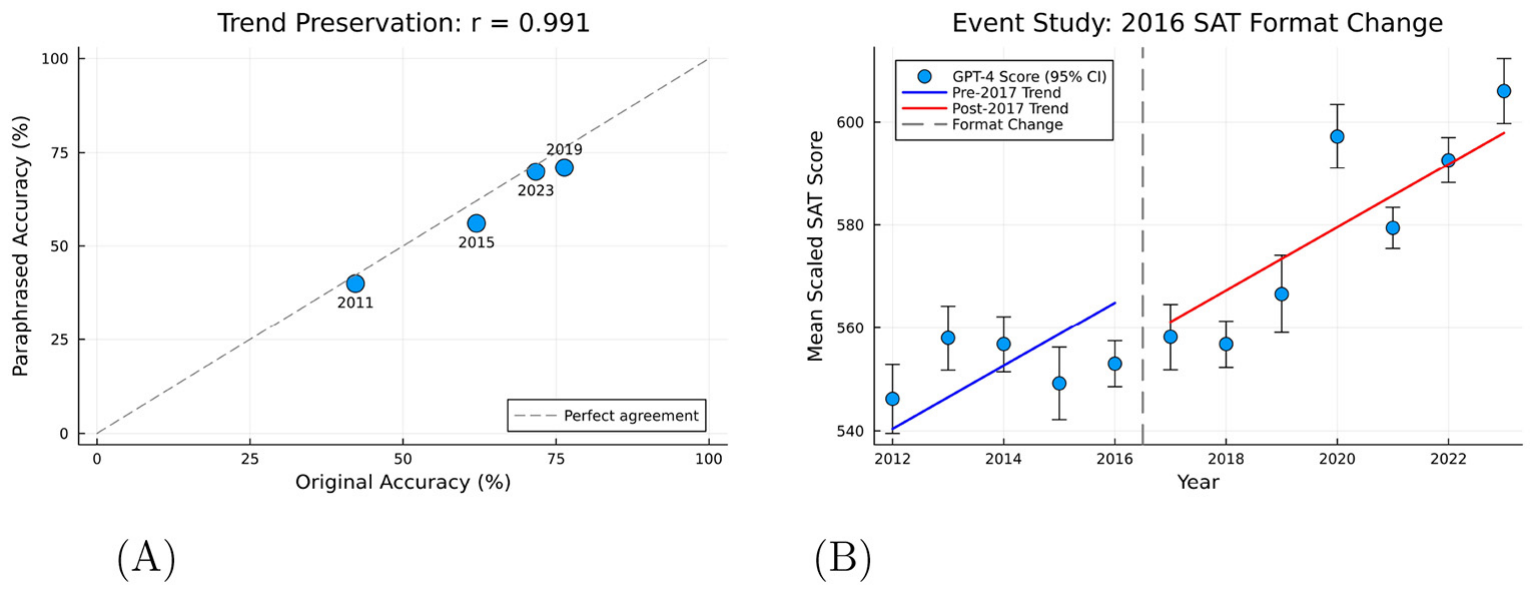
Robustness checks. **(A)** Shows the correlation between GPT-4 accuracy on original vs. paraphrased questions (*r* = 0.991), indicating temporal trends are preserved when surface text is altered. **(B)** Shows an event study at the 2016 SAT redesign; the jump coefficient is −9.82 (SE = 11.96) and is statistically insignificant (*p* = 0.433), confirming no discrete discontinuity at the format change. **(A)** Paraphrasing validation. **(B)** Event study: 2016 format change.

A key concern is whether GPT-4's improving performance reflects memorization of older SAT items that may appear in its training data (cutoff: September 2021). To address this, we administered paraphrased versions of 203 questions from years 2011, 2015, 2019, and 2023 to the same model. If memorization drove performance, paraphrasing should substantially reduce accuracy. Instead, as shown in Figure 7A, we find only a 3.9 percentage point drop in accuracy (64.0% to 60.1%), and crucially, the correlation between original and paraphrased accuracy across years is *r* = 0.991. This near-perfect preservation of the temporal trend strongly suggests GPT-4 is solving problems rather than retrieving memorized answers.

#### 4.3.2 2016 format change

The SAT underwent a major redesign in 2016. To test whether this format change confounds our difficulty estimates, we conducted an event study examining whether a discrete jump occurs at the 2016–2017 boundary. As shown in Figure 7B, the jump coefficient is statistically insignificant (β = −9.82, SE = 11.96, *p* = 0.433), indicating no discontinuity at the format change. The difficulty trend is smooth across the redesign, suggesting our results are not artifacts of format differences.

#### 4.3.3 Question type consistency

We examine whether the difficulty trend is driven by particular question types. Both multiple choice questions (MCQ) and student-produced response (grid-in) questions show nearly identical improvement: MCQ accuracy increased by 21.4 percentage points (43.6% to 65.0%) and grid-in accuracy increased by 21.4 percentage points (37.8% to 59.2%) from 2008 to 2023. This consistency across question formats supports the robustness of our difficulty estimates.

#### 4.3.4 Calculator vs. no calculator

The post-2016 SAT introduced a distinction between calculator-permitted and no-calculator sections. We assess the possibility that calculator policies might drive difficulty trends and find an insignificant performance difference: GPT-4 achieves a mean accuracy of 59.5% (SD = 4.8%) on Calculator sections versus 56.9% (SD = 5.8%) on No Calculator sections. The relatively small gap (~2.6 percentage points) in accuracy suggests that calculator policies are not a primary driver of the observed difficulty decline.

#### 4.3.5 Difficulty composition

Using a Random Forest classifier trained on pre-2014 College Board difficulty labels, we examine whether the composition of easy, medium, and hard questions changed over time. As shown in Figure 3, we find modest compositional drift: the proportion of hard questions shows a positive trend (*r* = 0.62), while easy questions show a negative trend (*r* = −0.44). However, the year-to-year variance is small (0.001–0.002), and there is no abrupt change at 2016, suggesting difficulty composition changes are gradual rather than discrete.

## 5 Discussion and conclusion

Standardized tests are routinely used to track performance trends, study inequality and downstream outcomes, but such comparability is reasonable only when the measurement instrument is stable or when test forms are credibly linked onto a common scale. In psychometric terms, linking and equating are intended to preserve score comparisons across administrations by adjusting for form difficulty, such that the same reported score corresponds to the same performance level (Kolen and Brennan, 2014; Holland and Dorans, 2006). However, when the conditions that justify these adjustments are stressed—because the assessment evolves, populations shift, or anchor information is unavailable—raw score trends can misstate changes in underlying performance (Michaelides, 2010). We study this comparability problem directly by introducing an “artificial test-taker” benchmark that makes difficulty drift measurable, and by using our benchmark as a transformed control to help separate changes in measured performance from changes in the measurement instrument.

Our primary result suggests that SAT Math difficulty is not constant over time, as measured by a fixed artificial participant exposed to year-specific items under invariant testing conditions. Two features of this benchmarking result are particularly consequential for interpretation. First, the benchmark changes are systematic rather than idiosyncratic, suggesting a time pattern consistent with drift rather than noise. Second, the benchmark is constructed by holding the test-taking protocol fixed, which clarifies that the estimated drift is driven by differences in test content rather than changes in the agent's “testing environment.” These findings align with the broader measurement literature that treats item drift as an empirical object to be monitored, not an assumption to be taken for granted (Guo et al., 2017; Lee and Lewis, 2021; Kang, 2023).

Our second key result uses the benchmark series to re-express observed student score trends after adjusting for test-difficulty. We find that the difficulty-adjusted decline in student performance is larger than as suggested by the raw score decline, implying that raw score trends that do not account for evolving exam difficulty may understate the decline in student performance. This mismeasurement is likely complicated by concurrent shifts in student participation due to widespread test-optional policies (Belasco et al., 2015; Bennett, 2021). More broadly, recent work on assessment in the age of generative AI emphasizes that the deployment of AI tools can both reveal and obscure inequities, depending on transparency, validation, and the interpretability of outputs (Swiecki et al., 2022; Kaldaras et al., 2024; Hao et al., 2024; Bulut et al., 2024). Finally, our results also indicate that difficulty-adjusted trends are not uniform across racial groups. The heterogeneity analysis underscores that comparability is not only a psychometric concern but can also affect how trends are perceived across sub-populations. In a setting where participation and incentives may shift differentially across groups over time, group-specific trajectories should be interpreted with care due to potential selection effects and changing composition of test-takers (Card and Rothstein, 2007).

Although in this study we perform supporting analysis to show question difficulty alignment between the LLM and the student test taker, the concept of alignment is still up for exploration and debate among Machine Learning and AI researchers (Bai et al., 2022; Ji et al., 2025). The LLM's lower accuracy on questions that student test-takers find difficult suggests that both the LLM and the test-takers perceive and are challenged by similar aspects of the questions. There likely exist factors that are influencing the LLM's performance that also affect human difficulty perception, such as question complexity and required reasoning skill. These factors can be further explored to understand the alignment between the LLM and the test-takers, but is beyond the scope of this study and may be an avenue for future research.

The LLMs in this study were not tuned to operate as a typical high school test taker; imbuing the LLMs with such a personality could offer stronger alignment. Further psychometric analysis of the LLM's performance on the SAT may provide insights into the detailed performance of the LLM. Multimodal AI models with visual inputs could analyze the SAT math section more thoroughly, these models were not available at the time of this study. These models can analyze image input but can misidentify the numerical data present in a graph or a chart, which has to be prompted separately adding to the complexity. While the National Assessment of Educational Progress (NAEP) has also noted a decline in math performance in recent years (National Center for Education Statistics, 2023) and a widening academic achievement gap between the rich and the poor (Reardon, 2018), the NAEP questions and the American College Testing (ACT) questions were not available to perform a similar comparative study. Despite these hindrances, our study provides a template for the use of AI as an artificial test-taker to develop counterfactuals for evaluating long-term trends in educational outcomes. As agentic and fine-tuned AI models continue to improve, they will enable increasingly better approximations of human test-taking behavior, ultimately yielding more robust counterfactuals for longitudinal educational research.

## Data Availability

The datasets presented in this study can be found in online repositories. The names of the repository/repositories and accession number(s) can be found below: https://github.com/krishnaveti/gpt_takes_sat_replication.

## Footnotes

1 Changes include no negative marking and restructuring of sections.

2 Given high performing multi-modal AI models that accept visual prompts were not available at the time of running this experiment, questions incorporating graphical elements (figures, charts, and diagrams) could not be transcribed for analysis. This exclusion ensured compatibility with the text-only input capabilities of the GPT-4 class models.

3 We were able to use preserved SATs from 2008 to 2011, which were not a part of our final results, but serve as training data for the machine learning model in this analysis.

4 Mean student score in 2012 was 543 after concordance and standard deviation was 117. We used the following formula: $\frac{\mu_{\text{Student},2012}-\mu_{\text{LLM,t}}}{\sigma_{\text{Student},2012}}$.

5 We used the following formula: $\frac{\mu_{\text{Student,t}}-\mu_{\text{LLM,t}}}{\sigma_{\text{Student,t}}}$.
