# Supplementary material for: Artificial test-takers as transformed controls: measuring SAT difficulty drift and student performance
Source: Front Artif Intell. 2026 Mar 16;9:1692465. doi: 10.3389/frai.2026.1692465 (PMC13033716; doi:10.3389/frai.2026.1692465)
Supplement: Supplementary file 1 [file Data_Sheet_1.pdf]

Appendix

Appendix A: Concordance Tables

The concordance tables provided below come directly from the College Board. We utilize these concordance tables in our paper for 2 types of score conversions: converting raw to scaled score and converting old to new score. The table below shows the conversion tables utilized by our study.

Appendix B: Data Sources

After collecting the PDF files, we transcribed each SAT exam PDF into a structured comma-separated values (CSV) file. MCQs present a set of possible answers, requiring the examinee to select the most appropriate option. Answer type allowed a one-line input from the examinee.

The College Board provides yearly reports with the population level SAT performance by the cohort of high school students taking the SAT exam. The data from these reports include average scores, total test takers and standard deviation in the scores for the mathematics test and the language and writing test. The concordance table provided by the College board requires rounding the average SAT score for exams in the *pre* period to the nearest multiple of 10 before it can be mapped to the average SAT score based on the concordance table. Due to this, the direct conversion of SAT scores using the concordance table has certain limitations. For example, if an average SAT score is 514 in the year 2009 and the score is 515 in the year 2010, 514 would be rounded to 510 and 515 would be rounded to 520, before the concordance table can be used. To avoid this problem, we used a simple linear regression model to regress average SAT scores before and after the conversion in the concordance table and then linearly interpolate the average SAT scores, which we call the Concordance SAT scores.

Negative marking

As previously stated, our study spans two eras, 2008-2016 known as *pre* and 2017-2023 known as *post* period. During *pre* period, the exams implemented negative marking, unlike the 2017-2023 period. In analyzing the GPT models' performance, we opted not to apply negative scoring for incorrect responses when converting from raw score to scaled score. This decision might initially suggest an inflated assessment of GPT performance in the *pre* period, as measured by scaled SAT scores. However, as our forthcoming sections demonstrate, incorporating negative marking for this

Table E.2: Concordance Tables for raw-to-scaled and pre-to-post score conversions

Post		Pre		Pre	Post
Raw Score	Scaled Score	Raw Score	Scaled Score	Scaled Score: Old	Scaled Score: New
58	800	54	800	200	200
57	790	53	790	210	220
56	780	52	760	220	230
55	760	51	740	230	250
54	750	50	720	240	260
53	740	49	710	250	280
52	730	48	700	260	300
51	710	47	690	270	310
50	700	46	680	280	330
49	690	45	670	290	340
48	680	44	660	300	350
47	670	43	650	310	360
46	670	42	640	320	360
45	660	41	640	330	370
44	650	40	630	340	380
43	640	39	620	350	390
42	630	38	610	360	400
41	620	37	600	370	410
40	610	36	590	380	420
39	600	35	590	390	430
38	600	34	580	400	440
37	590	33	570	410	450
36	580	32	560	420	460
35	570	31	550	430	470
34	560	30	540	440	480
33	560	29	540	450	490
32	550	28	530	460	500
31	540	27	520	470	510
30	530	26	510	480	510

Table E.3: Concordance Tables for raw-to-scaled and pre-to-post score conversions (continued)

Post		Pre		Pre	Post
Raw Score	Scaled Score	Raw Score	Scaled Score	Scaled Score: Old	Scaled Score: New
29	520	25	500	490	520
28	520	24	490	500	530
27	510	23	480	510	540
26	500	22	480	520	550
25	490	21	470	530	560
24	480	20	460	540	570
23	480	19	450	550	570
22	470	18	440	560	580
21	460	17	430	570	590
20	450	16	420	580	600
19	440	15	420	590	610
18	430	14	410	600	620
17	420	13	400	610	630
16	410	12	390	620	640
15	390	11	380	630	650
14	380	10	370	640	660
13	370	9	360	650	670
12	360	8	350	660	690
11	340	7	330	670	700
10	330	6	320	680	710
9	320	5	310	690	720
8	310	4	290	700	730
7	290	3	280	710	740
6	280	2	260	720	750
5	260	1	240	730	760
4	240	0	220	740	760
3	230	-1	200	750	770
2	210	-2	200	760	780
1	200			770	780
0	200			780	790
				790	800
				800	800

period would likely have amplified the performance discrepancy between the two periods. Therefore, our analysis likely presents a conservative estimate, potentially downplaying the actual performance gap.

Syllabus change

Although SAT math section did not experience a major change in structure and syllabus as compared to the verbal section, we must emphasize certain differences in exam content between the two periods. Before 2016, the key focus areas were arithmetic, numbers and operations, algebra, functions, geometry and data analysis. Starting 2016, SAT exam expanded the range of topics further and included questions related to trigonometry and complex numbers. Also, there was a greater focus on data analysis, graphs and word problems and less emphasis on geometry-related questions. Furthermore, starting 2016, students started received sub-scores for sections labeled as *Heart of Algebra*, *Passport to Advanced math* and *Problem Solving and Data Analysis*. There were no such subsections before 2016. Just by looking at the syllabus, one could argue that the math section became harder than before as it covered the number of topics covered increased, along with their difficulty level.

Data Sources

These reports provide comprehensive insights into the SAT performance of students on a state-by-state basis, enabling a detailed examination of trends and patterns in SAT scores across the United States. However, the availability of state-level SAT data prior to 2016 posed a unique challenge. To procure this historical data for the *pre* period (2008-2016), we used the Internet Archive and the National Center for Education Statistics. This digital archive, renowned for its extensive collection of web pages archived over time, proved instrumental in retrieving past state-level SAT reports not readily accessible on the College Board's current website. Utilizing this, we systematically sourced and compiled state-level SAT score reports for each year from 2008 to 2016. This approach of combining current and archived data sources ensures a comprehensive and continuous dataset spanning the entire duration of our study period.

Data Cleaning and Recentering

The distribution of the average scores for these school districts is provided in Figures 8a and 8b, which show the distribution of average scores before and after the recen-

tering using the concordance tables from the *pre* period to match the *post* format change period.

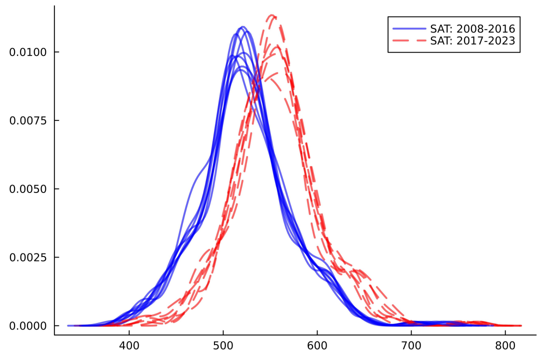

(a) Before Conversion

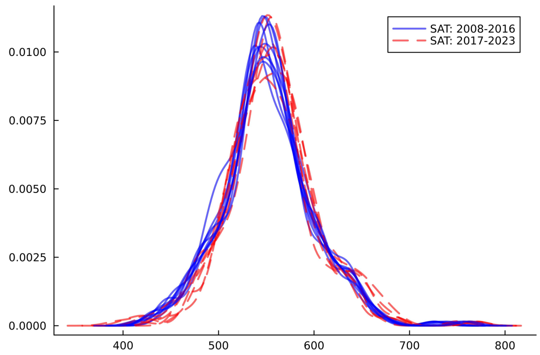

(b) After Conversion

Figure 8: SAT Score Distribution for School Districts in Massachusetts

Appendix C: Prompting

OpenAI provides access to their GPT series models through Application Programming Interface (API) endpoint. In this study, we used the most advanced model available GPT-4 . This model has been bench marked by OpenAI to be performing at the 89th percentile on SAT mathematics under certain prompting conditions as seen in OpenAI (2023a). For the purposes of this study, we employed a prompting strategy with explicit system level instructions to return the appropriate character letter corresponding to the correct answer having analyzed the question provided. Prompting strategies can affect the performance of the model as described by Nunes et al. (2023). Since, we are concerned with change in the LLM agent performance over time with reference to baseline, any idiosyncratic LLM effects and prompt effects cancel out. We employed a zero-shot prompt, by which, no examples solutions were provided to the model to facilitate answering the question. Further, we did not allow for chain-of-thought. The model was proscribed from sequentially reasons itself to the appropriate answer. The question was provided through the prompt and the model was asked to provide one character letter output for multiple choice questions and the appropriate numerical or equation output for the answer type questions. The prompting strategy is identical over all periods, ensuring there is no bias through the prompt. Since all prompts are independent API calls to the model, the model has no memory of previous questions. The model therefore is tasked to

answer each question independent of any other question or reference to the period from which the question is gathered. This ensured the comprehensive evaluation of the difficulty of the questions from appropriate periods.

The important parameters concerning this process are the temperature of the model and maximum token output limit. The temperature of the model governs the randomness of the output, the parameter when set to 0 provides a consistent and unwavering output from the LLM agent. This ensures the output from the LLM agent is deterministic and repetitive by always selecting the most probable token given the previous token. As the temperature is increased to higher levels, the agent has additional creative license to provide more abstract outputs. Since all LLM agents are predictive engines for the next token, higher temperature increases the probability of less likely token being selected as the output. For the purposes of our experiment and not to induce bias through the prompt temperature, we set it to 0. The maximum token output parameter limits the LLM agent's output. Since the agent in our experiment is required to choose the correct multiple choice option or provide straightforward numerical output, we limit the agent to 5 tokens, which is roughly 15-20 characters.

Our prompt template is shown in Listing 1, it encapsulates the strategy employed throughout the experiment. The LLM agent operates as an assistant to the user and to illicit a response from this assistant, two level of prompts are used. The system-level prompt is a high level instruction that the assistant is expected to follow verboten, this gives necessary context to the LLM agent and appropriately modulates its responses. For example, the LLM agent can be required to respond as a Shakespearean character through the system-level instructions. This is less useful in our case but provides the necessary platform to provide broad instructions to the agent about the task it is undertaking. The user-level prompt then provides the SAT question to the agent, the agent then responds to the output based on the temperature and maximum token output limit. Once these prompts and model parameters are held constant, we loop through the bootstrapped exams from each year as shown in Figure 2b. This ensures our agent is neutrally evaluating the SAT exam in the selected year while all other parameters are held constant.

Listing 1: SAT math Exam Instructions for GPT-4

```
@System-level prompt@
You are taking an SAT math exam which include multiple
    choice and answer type questions.
Determine the correct answer.
Choose only ONE 'character letter' response output
```

```
   corresponding to the correct answer from the options
   provided for multiple choice type.
Provide the appropriate numerical answer as required for
    the answer type question without any units of
   measurement.

@User-level prompt (for multiple-choice questions)@
You are provided with an SAT question enclosed in triple
    backticks, followed by multiple choice options.
``` <Question>
    <Options> ```
Please identify and return ONLY ONE letter character
   corresponding to the correct option.
Your response output should only be ONE character letter
    .

@User-level prompt (for numerical answer questions)@
You are provided with an SAT question of numerical type,
    enclosed in triple backticks. Please determine and
   return the correct numerical value or mathematical
   expression.
``` <Question> ```
WARNING: Do not provide any explanations, calculations,
   units of measurement, or additional outputs.
```

Appendix D: Other LLMs

To ensure the robustness of our findings and to validate that our results are not subject to the specific LLM used, we conducted additional tests using different versions of the LLM. We utilized the GPT-4 April update and another LLM, Claude 3.5 Sonnet, for this purpose. GPT-4 Turbo received an update on April 9th, 2024. This update by OpenAI majorly improved the model performance (OpenAI, 2023b). The results in the main text are from GPT-4 January update, and we provide the results from GPT-4 April update. The results show a similar trend in the decline of student performance over time. The decline in SAT scores is 113 points at the national level in 2023 compared to 2008. The decline in SAT scores is 72 points at the Massachusetts level in 2023 compared to 2008. Additionally, it can be noted through Figure 9 that the performance of GPT-4 April tracks the GPT-4 January in

evaluating underling SAT. The results are consistent with the main text and show a decline in SAT math difficulty over time.

Next, we utilized Claude 3.5 Sonnet, a different LLM, to evaluate the SAT exams. Claude 3.5 Sonnet is a LLM developed by Anthropic, a U.S based artifical intelligence (AI) company. We used this model as model benchmarking performed by Anthropic show similar performance results compared to OpenAI's GPT-40 model (Anthropic, 2024). The results from Claude 3.5 Sonnet are also provided in Figure 9. In the figure, we can clearly see that the initial and end point performance of Claude 3.5 Sonnet on the SAT exams is consistent with the performance of GPT-4 January and GPT-4 April update. The overall trend of declining SAT scores from 2008 to 2023 was replicated by these runs, further strengthening our claim. This robustness test demonstrates that our findings are consistent across different LLMs, provided that the intelligence demonstrated by LLMs is comparable, and are not an artifact of the specific LLM used in the initial analysis.

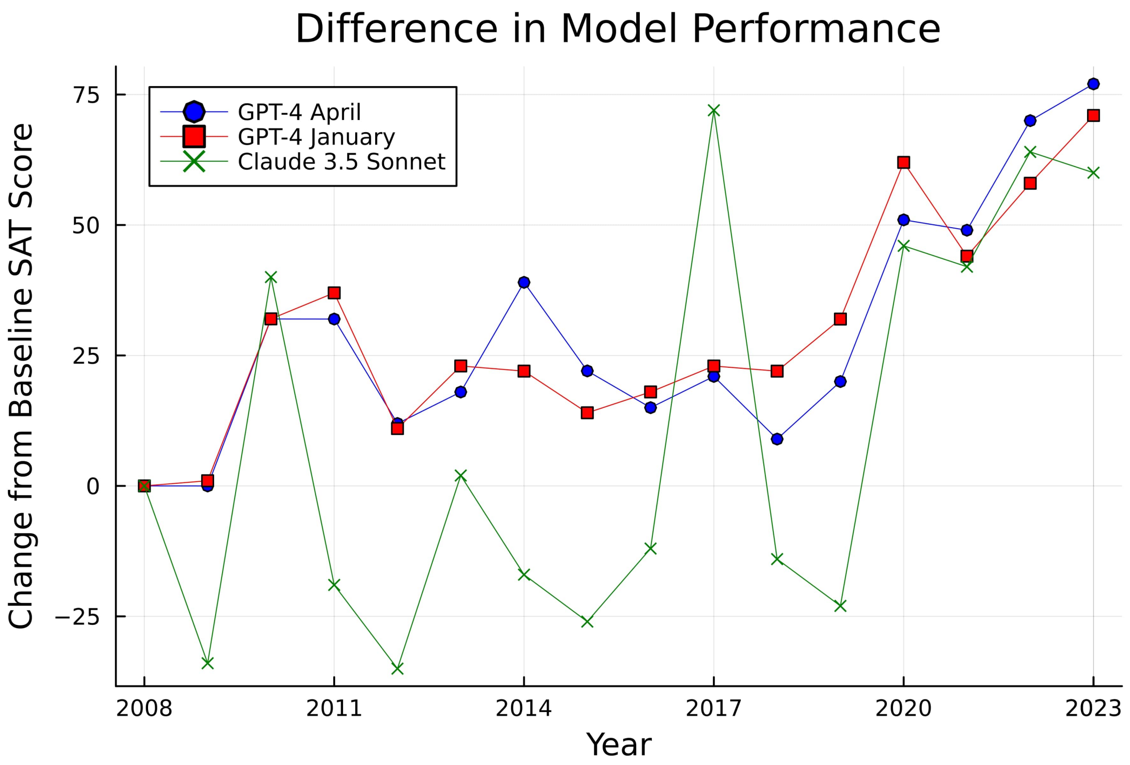

Figure 9: Change in Performance of GPT-4 April and GPT-4 January

Appendix E: Additional Tables and Figures

Table E.4: Yearly Performance of LLM Agent by Question Type

Year	All Questions		Multiple Choice only		Answer Type only	
	Estimate	S.E.	Estimate	S.E.	Estimate	S.E.
2008	0.469	(0.008)	0.495	(0.009)	0.378	(0.018)
2009	0.473	(0.007)	0.459	(0.008)	0.523	(0.013)
2010	0.548	(0.006)	0.525	(0.007)	0.630	(0.013)
2011	0.560	(0.008)	0.555	(0.009)	0.578	(0.011)
2012	0.494	(0.008)	0.493	(0.009)	0.500	(0.014)
2013	0.523	(0.008)	0.546	(0.009)	0.445	(0.013)
2014	0.519	(0.006)	0.543	(0.008)	0.435	(0.013)
2015	0.502	(0.008)	0.517	(0.010)	0.448	(0.015)
2016	0.508	(0.005)	0.516	(0.006)	0.478	(0.007)
2017	0.574	(0.007)	0.603	(0.008)	0.474	(0.015)
2018	0.569	(0.005)	0.592	(0.006)	0.492	(0.009)
2019	0.592	(0.008)	0.630	(0.008)	0.462	(0.018)
2020	0.657	(0.007)	0.654	(0.008)	0.669	(0.015)
2021	0.619	(0.004)	0.578	(0.006)	0.762	(0.005)
2022	0.648	(0.005)	0.672	(0.006)	0.566	(0.011)
2023	0.675	(0.007)	0.699	(0.007)	0.592	(0.018)

Notes: This table reports the average proportion of questions correctly answered by the LLM agent each year. The estimates and standard errors (S.E.) for the proportion of questions correctly answered by question type are provided. The estimates represent the ratio of correctly answered questions to the total number of questions in that category of question type.

Table E.5: Asian, Black and White Student SAT Score Changes

Year	Asian		Black		White	
	Estimate	S.E.	Estimate	S.E.	Estimate	S.E.
2009	3.595	(4.764)	-1.326	(4.74)	-2.493	(4.73)
2010	-22.978	(4.255)	-29.926	(4.204)	-33.027	(4.203)
2011	-24.386	(4.797)	-36.367	(4.818)	-39.425	(4.819)
2012	1.832	(5.099)	-9.17	(5.039)	-12.268	(5.001)
2013	-7.993	(4.945)	-19.935	(4.881)	-26.079	(4.899)
2014	-5.761	(4.599)	-18.729	(4.609)	-24.826	(4.623)
2015	1.808	(5.195)	-12.163	(5.177)	-17.219	(5.179)
2016	1.993	(4.331)	-18.94	(4.295)	-22.032	(4.329)
2017	-19.174	(4.988)	-18.104	(4.88)	-35.219	(4.969)
2018	5.208	(4.371)	-15.736	(4.318)	-29.852	(4.351)
2019	-2.59	(5.358)	-31.52	(5.423)	-43.61	(5.454)
2020	-38.206	(4.904)	-65.152	(4.859)	-80.246	(4.873)
2021	-10.403	(4.171)	-44.337	(4.179)	-59.447	(4.248)
2022	-32.568	(4.31)	-62.526	(4.347)	-79.617	(4.302)
2023	-52.988	(4.924)	-86.969	(4.898)	-103.99	(4.892)

Table E.6: Male and Female Student SAT Score Changes

Year	Male		Female	
	Estimate	S.E.	Estimate	S.E.
2009	-0.385	(4.766)	-2.353	(4.725)
2010	-30.991	(4.248)	-32.007	(4.207)
2011	-39.401	(4.847)	-37.417	(4.84)
2012	-12.23	(5.051)	-12.199	(5.055)
2013	-24.989	(4.923)	-23.996	(4.891)
2014	-24.796	(4.688)	-22.831	(4.581)
2015	-20.182	(5.176)	-18.197	(5.152)
2016	-26.988	(4.35)	-23.984	(4.292)
2017	-46.147	(4.863)	-36.227	(4.925)
2018	-40.797	(4.357)	-28.773	(4.299)
2019	-55.57	(5.359)	-41.587	(5.356)
2020	-92.155	(4.91)	-75.156	(4.913)
2021	-68.379	(4.234)	-54.405	(4.151)
2022	-88.565	(4.349)	-74.586	(4.293)
2023	-117.006	(4.897)	-100.039	(4.876)

Table E.7: Yearly National and Massachusetts SAT Score Changes after removing post-COVID years

Year	National		Massachusetts	
	Estimate	S.E.	Estimate	S.E.
2009	-1.831	(5.495)	1.052	(3.208)
2010	-32.471	(5.495)	-27.776	(3.208)
2011	-39.792	(5.495)	-34.448	(3.208)
2012	-13.357	(5.495)	-6.112	(3.208)
2013	-25.980	(5.495)	-20.404	(3.208)
2014	-23.271	(5.495)	-17.173	(3.208)
2015	-17.063	(5.495)	-10.441	(3.208)
2016	-18.784	(5.495)	-13.114	(3.208)
2017	-33.416	(5.495)	-15.897	(3.208)
2018	-31.643	(5.495)	-11.861	(3.208)
2019	-46.522	(5.495)	-26.236	(3.208)
2020	-83.749	(5.495)	-54.599	(3.208)

Notes: This table reports the decline after removing post-COVID years. The national sample represented by the unweighted state SAT data.

Appendix G: Question Embeddings and Difficulty Alignment

The difficulty of the questions is predicted using a Random Forest Classifier trained on the question embeddings and question location within the section as features. The Table E.8 provides the classifier accuracy for the test sample. The standard error is calculated using the proportion of predicted difficulty rating for questions in the bootstrapped SATs. It does not account for the uncertainty in the classifier for the unseen data between 2015 and 2023.

Table E.8: Confusion Matrix: Classification Accuracy - Test Sample

**Predicted/Ground Truth**	**Easy**	**Medium**	**Hard**
**Easy**	130	15	1
**Medium**	17	61	23
**Hard**	1	4	36
